# Supplementary figures and images for: Evaluation of miniaturized Illumina DNA preparation protocols for SARS-CoV-2 whole genome sequencing
Source: PLoS One. 2023 Apr 26;18(4):e0283219. doi: 10.1371/journal.pone.0283219 (PMC10132692; doi:10.1371/journal.pone.0283219)

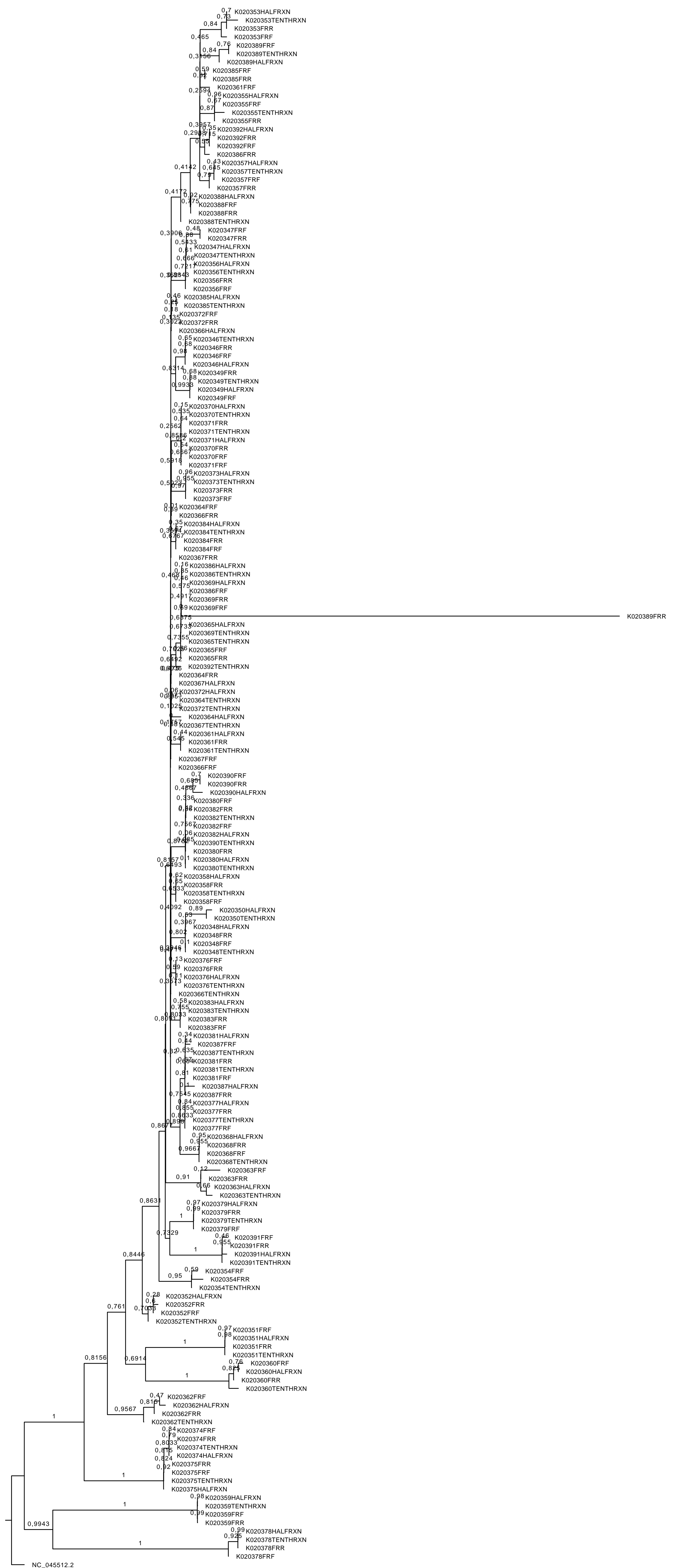

5.0E-4

Supplement: S1 Fig — (PDF) [file pone.0283219.s003.pdf]
