# Supplementary material for: Evaluation of miniaturized Illumina DNA preparation protocols for SARS-CoV-2 whole genome sequencing
Source: PLoS One. 2023 Apr 26;18(4):e0283219. doi: 10.1371/journal.pone.0283219 (PMC10132692; doi:10.1371/journal.pone.0283219)
Supplement: Attachment — Submitted filename: Response to reviewers.docx [file pone.0283219.s004.docx]

Journal requirements:

When submitting your revision, we need you to address these additional requirements.

1.  Please ensure that your manuscript meets PLOS ONE's style requirements, including those for file naming. The PLOS ONE style templates can be found at

<https://journals.plos.org/plosone/s/file?id=wjVg/PLOSOne_formatting_sample_main_body.pdf>  and

<https://journals.plos.org/plosone/s/file?id=ba62/PLOSOne_formatting_sample_title_authors_affiliations.pdf>

2. Please provide additional details regarding participant consent. In the ethics statement in the Methods and online submission information, please ensure that you have specified (1) whether consent was informed and (2) what type you obtained (for instance, written or verbal, and if verbal, how it was documented and witnessed). If your study included minors, state whether you obtained consent from parents or guardians. If the need for consent was waived by the ethics committee, please include this information.

If you are reporting a retrospective study of medical records or archived samples, please ensure that you have discussed whether all data were fully anonymized before you accessed them and/or whether the IRB or ethics committee waived the requirement for informed consent. If patients provided informed written consent to have data from their medical records used in research, please include this information.

The study did not involve human subjects, therefore informed consent was not required. As mentioned in the manuscript lines lines 211-218, remnant samples were used from the National Health Laboratory Service as part of a SARS-CoV-2 genomic surveillance programme. Ethical approval for genomic surveillance performed in this study was obtained from the university of Kwazulu Natal IRB approval number BREC/00001510/2020.

3. Thank you for stating the following financial disclosure:

“TdO- Rockefeller Foundation (HTH 017),  Abbott Pandemic Defense Coalition (APDC), the African Society for Laboratory Medicine, the National Institute of Health USA (U01 AI151698) for the United World Antivirus Research Network (UWARN) and the INFORM Africa project through IHVN (U54 TW012041), H3BioNet Africa (Grant # 2020 HTH 062), the South African Department of Science and Innovation (SA DSI) and the South African Medical Research Council (SAMRC) under the BRICS JAF #2020/049 and the World Bank (TF0B8412).

JG- South African Medical Research Council (MRC SIR-HIVDR POC)”

Please state what role the funders took in the study.  If the funders had no role, please state: "The funders had no role in study design, data collection and analysis, decision to publish, or preparation of the manuscript."

"The funders had no role in study design, data collection and analysis, decision to publish, or preparation of the manuscript."

If this statement is not correct you must amend it as needed.

Please respond by return e-mail so that we can amend your financial disclosure and competing interests on your behalf.

4. We note that you have stated that you will provide repository information for your data at acceptance. Should your manuscript be accepted for publication, we will hold it until you provide the relevant accession numbers or DOIs necessary to access your data. If you wish to make changes to your Data Availability statement, please describe these changes in your cover letter and we will update your Data Availability statement to reflect the information you provide.

All the raw sequence data generated for this study has been made publicly available on the NCBI short read archive (SRA) under project no. [PRJNA926488](https://www.ncbi.nlm.nih.gov/bioproject/PRJNA926488).

Reviewers' comments:

Reviewer's Responses to Questions

**Comments to the Author**

1. Is the manuscript technically sound, and do the data support the conclusions?

The manuscript must describe a technically sound piece of scientific research with data that supports the conclusions. Experiments must have been conducted rigorously, with appropriate controls, replication, and sample sizes. The conclusions must be drawn appropriately based on the data presented.

Reviewer #1: Partly

Reviewer #2: Yes

Reviewer #3: Yes

Reviewer #4: Yes

2. Has the statistical analysis been performed appropriately and rigorously?

Reviewer #1: No

Reviewer #2: Yes

Reviewer #3: Yes

Reviewer #4: Yes

3. Have the authors made all data underlying the findings in their manuscript fully available?

The [PLOS Data policy](http://www.plosone.org/static/policies.action#sharing) requires authors to make all data underlying the findings described in their manuscript fully available without restriction, with rare exception (please refer to the Data Availability Statement in the manuscript PDF file). The data should be provided as part of the manuscript or its supporting information, or deposited to a public repository. For example, in addition to summary statistics, the data points behind means, medians and variance measures should be available. If there are restrictions on publicly sharing data—e.g. participant privacy or use of data from a third party—those must be specified.

Reviewer #1: No

Reviewer #2: Yes

Reviewer #3: No

Reviewer #4: No

4. Is the manuscript presented in an intelligible fashion and written in standard English?

PLOS ONE does not copyedit accepted manuscripts, so the language in submitted articles must be clear, correct, and unambiguous. Any typographical or grammatical errors should be corrected at revision, so please note any specific errors here.

Reviewer #1: Yes

Reviewer #2: Yes

Reviewer #3: Yes

Reviewer #4: Yes

5. Review Comments to the Author

Reviewer #1: 
This manuscript by Sureshnee Pillay et al. describes a new (umpteenth) miniaturization method for preparing libraries for sequencing.

This manuscript is severely lacking in novelty to be published. In addition, the statistical comparisons are missing, for the most part, and the manuscript can be corrected according to my recommendations below.
Line 104/284: "to date"/"at the time" should be replaced by the date.
"et al." should be italicized.

We have updated the manuscript according to the reviewers recommendation. We added the appropriate timing. See Page 4, line 87 and page 14 line 487-488..

Global: the manuscript should be turned to the passive.

As recommended by the reviewer, we have changed the active sections of the manuscript to passive, for instance;

page 5, lines 166-167, have changed from “As a result, protocols have been modified to maximize reagent use” to “ As a result of limited supplies, protocol modification has been adopted as means to maximize reagent use (10) and drive down sequencing costs”.

page 6, lines 187-188, have changed from “”we sought to adapt our library preparation protocol into a lower-cost, streamlined protocol to “ in this study, an adaption of our library preparation protocol into a lower cost, streamlined protocol is described”.

Method: how was the number of samples to be sequenced determined (other than 48*2=96 for the indexes I guess?). Explanation of how the libraries are matched for sequencing is missing.

Yes, the reviewer is right, to manage the cost of sequencing, we split for methods across two sequencing runs. Each run accounted for 2 methods each having 48 samples. Importantly, the method of interest in this manuscript has since been used across several sequencing runs successfully which gives us further confidence in its performance and accuracy.

Present (at least in appendix) the primers used.

The table of primers has been added as supplementary table 1.

Websites must be referenced in the bibliography and not in the body of the text.

Please see below:

P13-Line 447

P24- line 704

The experience of the operator has nothing to do in the body of the methods (possibly in the discussions, and even then it is not very useful ...)

We agree with the reviewer and have removed this line from the text.

How do the authors explain the difference in quality of a quarter protocol, when a tenth protocol worked better? Also, there is a lack of statistical comparisons to get an idea.

In this study, we tested the full reaction as per manufactures instructions against the rapid protocol for the full, half and tenth reactions. We did not test the quarter. Although all the reactions performed well, in this manuscript we focused on the 10th reaction as it offered the most saving on the laboratory reagents and produced the sequences that were closest to the full reaction. We however, find this a interesting observation that would require validating across additional runs as we only tested the half reaction in one sequencing run. We have highlighted this in the limitations section of our manuscript and could be a basis for a larger validation study.

The sequences produced must be published (NCBI?) to be able to validate the results obtained.

As requested, all the raw sequence data generated for this study has been made publicly available on the NCBI short read archive (SRA) under project no. [PRJNA926488](https://www.ncbi.nlm.nih.gov/bioproject/PRJNA926488).

Reviewer #2: 
This article is a research article that describes SARS-CoV-2 miniaturized sequencing protocol. CODVI-19 related research is of importance, especially an effective and cheap way of DNA sequence for future potential variants. I recommended acceptance after major revision.

1. Abstract should be a single paragraph instead of including four bullet points, since Introduction, Methods, Results, and Conclusion will be discussed in the main text. My suggestion is to polish and merge them to one paragraph less than 300 words.

The abstract has been merged as per the reviewers recommendation: Page 1, lines 43-70.

2. Lack of comprehensive literature review on current DNA sequencing technology using other methods.

Content added: page 4-5: line 90-162.

3. Figure quality is low. My suggestion is to increase the resolution and enhance the pictures. Also, bad color selections in figure 3, since Orange and Beige colors are not easy to tell the difference to many audiences, including me. My suggestion is to use a high-contrast color combination in figure design, for example the color choice in figure 4. Actually, keeping the color selection consistently is a good strategy to help audience understand the whole article by receiving the same color pattern information.

Figure 3. has been re-done with different colour selections, thus enhancing the quality, as per the reviewers recommendations.

4. Some spelling and grammar mistakes. My suggestion is to perform more proof-reading.

This has been addressed.

Reviewer #3: 
The authors describe three modified sequencing protocols using Illumina platforms with the aim to reduce time, reagents and cost for SARS-CoV-2 surveillance. Some points need to be addressed.

1. Sequence data (FastQ) obtained by the three protocols need to be free accessible to the scientific community. I strongly suggest depositing these data on free accessible databases (es. Sequence Read Archive).

As suggested by the reviewer, all the raw sequence data generated for this study has been made publicly available on the NCBI short read archive (SRA) under project no. [PRJNA926488](https://www.ncbi.nlm.nih.gov/bioproject/PRJNA926488).

2. A phylogenetic analysis based on a Maximum likelihood approach that highlights the reproducibility of the three protocols by comparing the sequence data needs to be addressed.

A maximum likelihood phylogenetic tree has been inferred in IQTREE2 using the GTR evolutionary model and 100 boostrap replicates. The inferred tree is available as supplementary figure 1. As shown in the tree, we see a high level of congruence and similarity between the sequences from each method.

3. It is important to provide evidence of reproducibility of the protocols across lineages (Omicron sublineages as latest). At this regard, the authors did not mention the lineage of the 47 samples used.

This is information is provided in Table S1 and discussed on page 17: lines 547-550.

4. The authors should discuss the advantages that these methods can have in other settings, like other emerging and re-emerging pathogens surveillance (es. Ebola).

Page 20: lines 635-643.

Reviewer #4:

The study evaluation of different volume of library for SARS-CoV-2. Detailed library preparation kit is not provided, Nextera XT, or DNA flex ???

Illumina DNA Prep- line 231

The detailed procedure/SOP needs to be deposited on to the Protocols.IO platform and link it to the manuscript.

Line 231- [dx.doi.org/10.17504/protocols.io.n92ldpp8nl5b/v1](http://dx.doi.org/10.17504/protocols.io.n92ldpp8nl5b/v1)

The NGS raw fastq files needs to be deposited to PubMed.

As suggested by the reviewer, all the raw sequence data generated for this study has been made publicly available on the NCBI short read archive (SRA) under project no. [PRJNA926488](https://www.ncbi.nlm.nih.gov/bioproject/PRJNA926488).

Lines 179-206, which kit used in the study, please provide the detailed information on it.

Nextera DNA Prep_line 231.

Table 2, how much of amplicon needed in stead of volume need also to be provided.

DNA was used neat and undiluted.

Lines 208-233, please provide a table to summarize the difference of non-rapid and rapid method process.

Information is provided in Table 3.

6. PLOS authors have the option to publish the peer review history of their article ([what does this mean?](https://journals.plos.org/plosone/s/editorial-and-peer-review-process#loc-peer-review-history)). If published, this will include your full peer review and any attached files.


If you choose “no”, your identity will remain anonymous but your review may still be made public.


**Do you want your identity to be public for this peer review?** For information about this choice, including consent withdrawal, please see our [Privacy Policy](https://www.plos.org/privacy-policy).

Reviewer #1: No

Reviewer #2: No

Reviewer #3: No

Reviewer #4: No

[NOTE: If reviewer comments were submitted as an attachment file, they will be attached to this email and accessible via the submission site. Please log into your account, locate the manuscript record, and check for the action link "View Attachments". If this link does not appear, there are no attachment files.]

While revising your submission, please upload your figure files to the Preflight Analysis and Conversion Engine (PACE) digital diagnostic tool, <https://pacev2.apexcovantage.com/>. PACE helps ensure that figures meet PLOS requirements. To use PACE, you must first register as a user. Registration is free. Then, login and navigate to the UPLOAD tab, where you will find detailed instructions on how to use the tool. If you encounter any issues or have any questions when using PACE, please email PLOS at [figures@plos.org](mailto:figures@plos.org). Please note that Supporting Information files do not need this step.
